# Effects of realistic sheep elbow kinematics in inverse dynamic simulation

**DOI:** 10.1371/journal.pone.0213100

**Published:** 2019-03-05

**Authors:** Baptiste Poncery, Santiago Arroyave-Tobón, Elia Picault, Jean-Marc Linares

**Affiliations:** Aix Marseille Univ, CNRS, ISM, Marseille, France; University of Memphis, UNITED STATES

## Abstract

Looking for new opportunities in mechanical design, we are interested in studying the kinematic behaviour of biological joints. The real kinematic behaviour of the elbow of quadruped animals (which is submitted to high mechanical stresses in comparison with bipeds) remains unexplored. The sheep elbow joint was chosen because of its similarity with a revolute joint. The main objective of this study is to estimate the effects of elbow simplifications on the prediction of joint reaction forces in inverse dynamic simulations. Rigid motions between humerus and radius-ulna were registered during full flexion-extension gestures on five cadaveric specimens. The experiments were initially conducted with fresh specimens with ligaments and repeated after removal of all soft tissue, including cartilage. A digital image correlation system was used for tracking optical markers fixed on the bones. The geometry of the specimens was digitized using a 3D optical scanner. Then, the instantaneous helical axis of the joint was computed for each acquisition time. Finally, an OpenSim musculoskeletal model of the sheep forelimb was used to quantify effects of elbow joint approximations on the prediction of joint reaction forces. The motion analysis showed that only the medial-lateral translation is sufficiently large regarding the measuring uncertainty of the experiments. This translation assimilates the sheep elbow to a screw joint instead of a revolute joint. In comparison with fresh specimens, the experiments conducted with dry bone specimens (bones without soft tissue) provided different kinematic behaviour. From the results of our inverse dynamic simulations, it was noticed that the inclusion of the medial-lateral translation to the model made up with the mean flexion axis does not affect the predicted joint reaction forces. A geometrical difference between the axis of the best fitting cylinder and the mean flexion axis (derived from the motion analysis) of fresh specimens was highlighted. This geometrical difference impacts slightly the prediction of joint reactions.

## Introduction

The natural evolution of biological joints (articular geometry, material properties and mechanisms of tissue repair) has generated efficient joints in terms of mechanical performance. Looking for new opportunities in mechanical design, we are interested in studying the kinematic behaviour of biological joints. Unlike dorsomobiles, dorsostable mammals are able to stand for long periods. This study focuses on the kinematic behaviour of the elbow of a dorsostable mammal. This joint is interesting because of its similarity with a revolute joint.

Loads on biological joints are usually determined by inverse dynamic simulation using musculoskeletal models [1–4]. In these models, however, biological joints are usually simplified and considered as perfect joints (geometrically perfect and frictionless) [5]. In particular, the elbow joint is commonly modelled by a revolute (or hinge) joint [1,2,4,6]. However, the effects of such simplifications in simulations of animal locomotion remain unexplored.

In the literature, several methods have been used to estimate joint parameters for musculoskeletal modelling. Some studies used anatomical landmarks [7–9] or contact surfaces best fit [10,11] to estimate joint rotation axis. In a different approach, [12–15] used movement analysis based on markers tracking for the same purposes. Some of these studies were conducted *in vivo*, on humans or animals [12,16]. However, their main drawback is that soft tissue artifact strongly impacts the accuracy of the measurement [16]. Similar studies, such as those of Lynch, S. et Al. [14] and Cuddy, L. [17], were performed *in vitro* with markers fixed directly on the bones, avoiding these measuring perturbations.

For representing the kinematics of synovial joints in musculoskeletal modelling different models have been used. The simplest one considers only one degree of freedom (DoF) (flexion-extension), representing a perfect revolute joint located in the sagittal plan [16,18,19]. Other studies [20,21] implemented a 2 DoF model considering flexion-extension and pronation-supination movements. In [22,23], a 3 DoF model has been used considering additionally axis 2D displacements of the flexion axis in the sagittal plane (3 DoF: flexion-extension, posterior-anterior translation and proximo-distal translation). A 5 DoF has also been used, introducing adduction-abduction and internal-external rotation [8,24]. The most complex models also take into account the axial translation, defining a 6 DoF model [25–29]. These models can be mathematically represented using, for example, screw theory [12,24,30]. Most of these studies are focused on the knee joint biomechanics, and those dealing with the elbow involve only human beings. Therefore, the kinematic behaviour of the elbow of quadruped animals remains fairly unexplored.

In response to these voids in the literature, the objectives of this work are: i) to study the kinematics of the sheep elbow, ii) to evaluate the influence of the ligaments and cartilage on the joint kinematics and iii) to estimate the effects of elbow simplifications on the prediction of joint reaction forces. To reach these goals, rigid motions between humerus and radius-ulna were registered during full flexion-extension gestures on cadaveric specimens. A digital image correlation system was used for tracking optical markers fixed on the bones. This system provides a good measurement accuracy (0.002 mm) while allowing high frequency acquisition throughout the movement. Then, the instantaneous helical axis of the joint was computed for each acquisition time. This analysis was also performed on the same specimens after removing soft tissue and cartilage, with the goal of determining if dry bone specimens can be used for kinematic analysis in biomechanics, which might be interesting in studies of extinct taxa, for example [31–33]. Finally, an OpenSim musculoskeletal model of the sheep forelimb was used to quantify effects of elbow joint approximations on the prediction of joint reaction forces.

## Materials and methods

**Fig 1:**
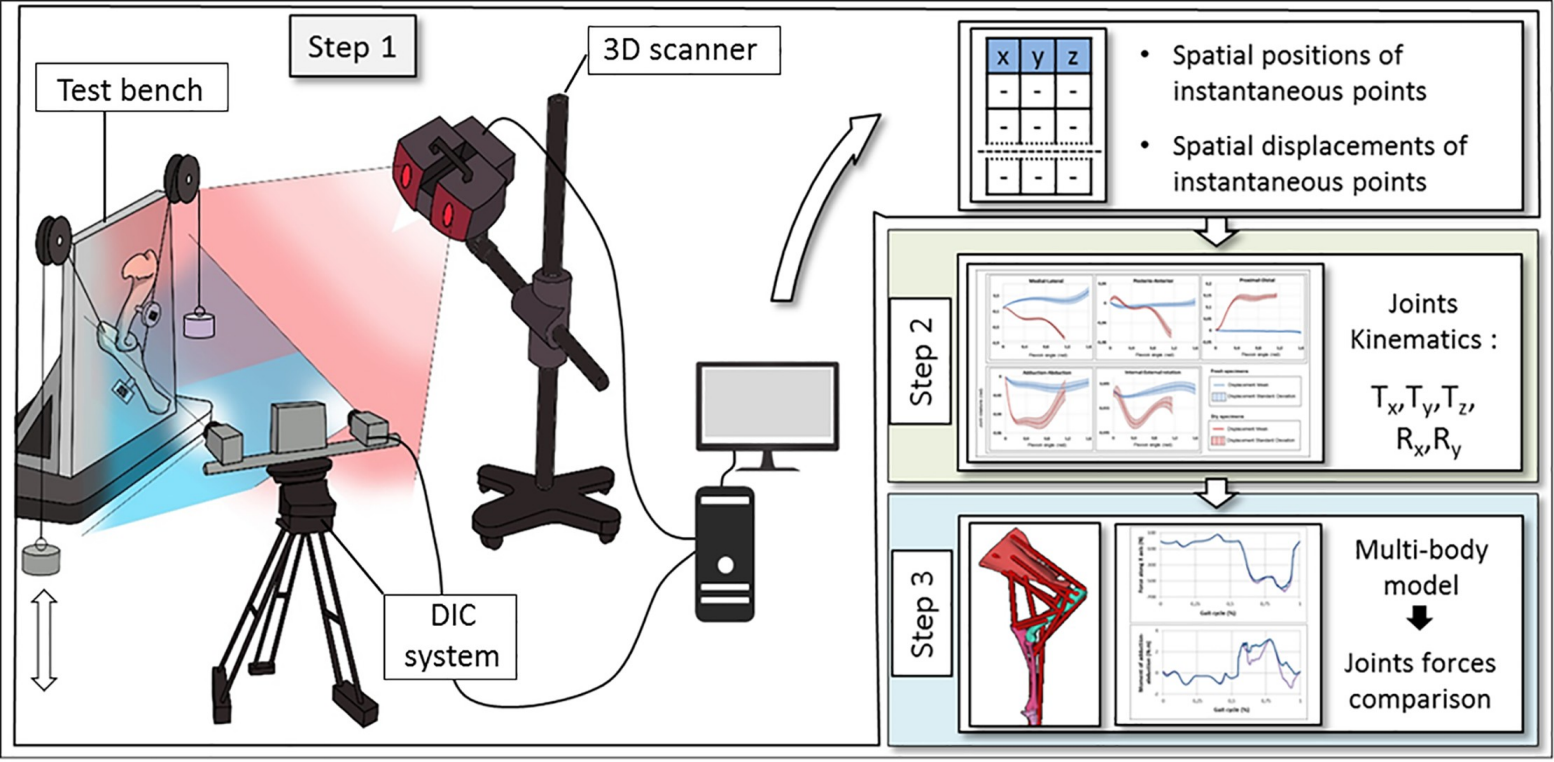
Process for data acquisition and post processing.

In this section, the procedure used to determine the effects of joint simplification in musculoskeletal dynamic simulation is described. This procedure was divided in three steps as shown in Fig 1. The first one was focused on the measurement of kinematics during a flexion-extension gesture on cadaveric specimens (sheep elbow). The measurement was done using a digital image correlation (DIC) system; bones were also digitized using a 3D optical scanner. The second step consisted on data post-processing to develop a realistic kinematic model of the elbow. The third step introduced the developed kinematic model in a musculoskeletal model of the whole sheep forelimb for performing inverse dynamic simulations of a gait trial. These three steps are detailed in the rest of this section.

### Data acquisition of elbow flexion motion

**Fig 2:**
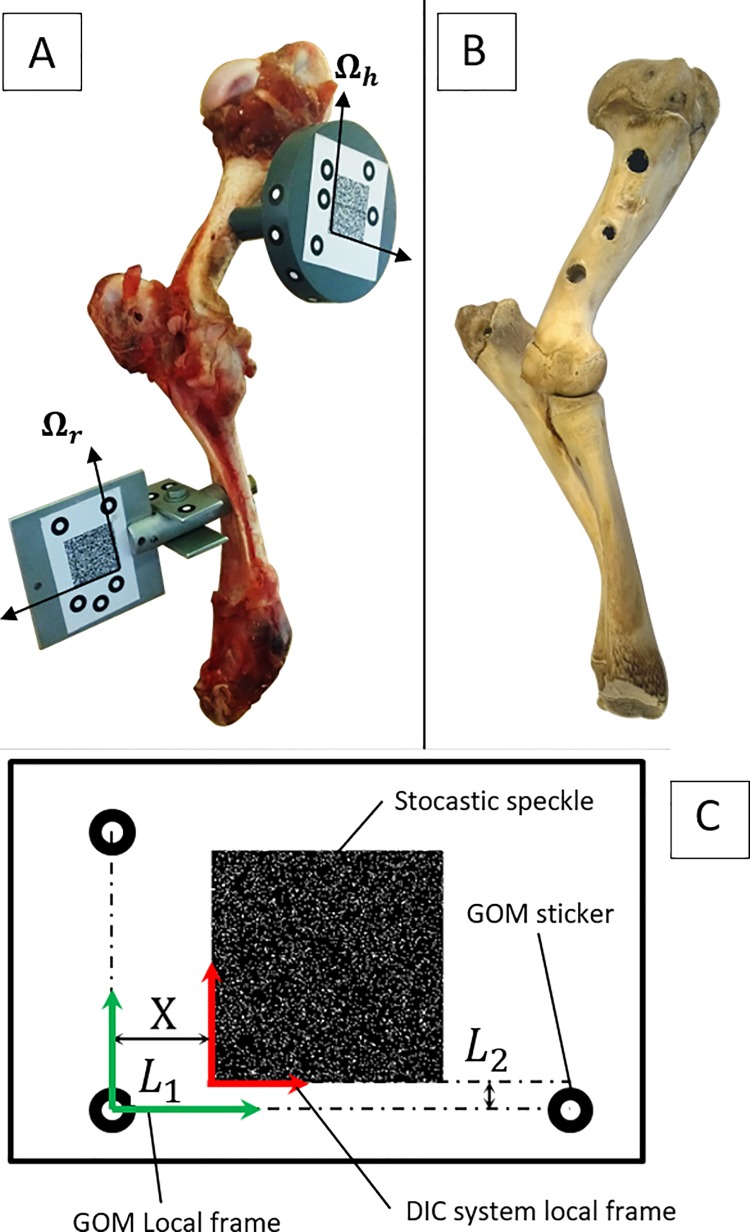
Sheep elbow specimens. (A) Fresh sheep elbow specimen with ligaments and synovial capsule, equipped with tracking clusters. (B) Sheep elbow specimen after removal of all soft tissue by boiling. (C) Composition of tracking clusters: Location of the speckle tracking mark relative to the 3D scanner frame.

**Fig 3:**
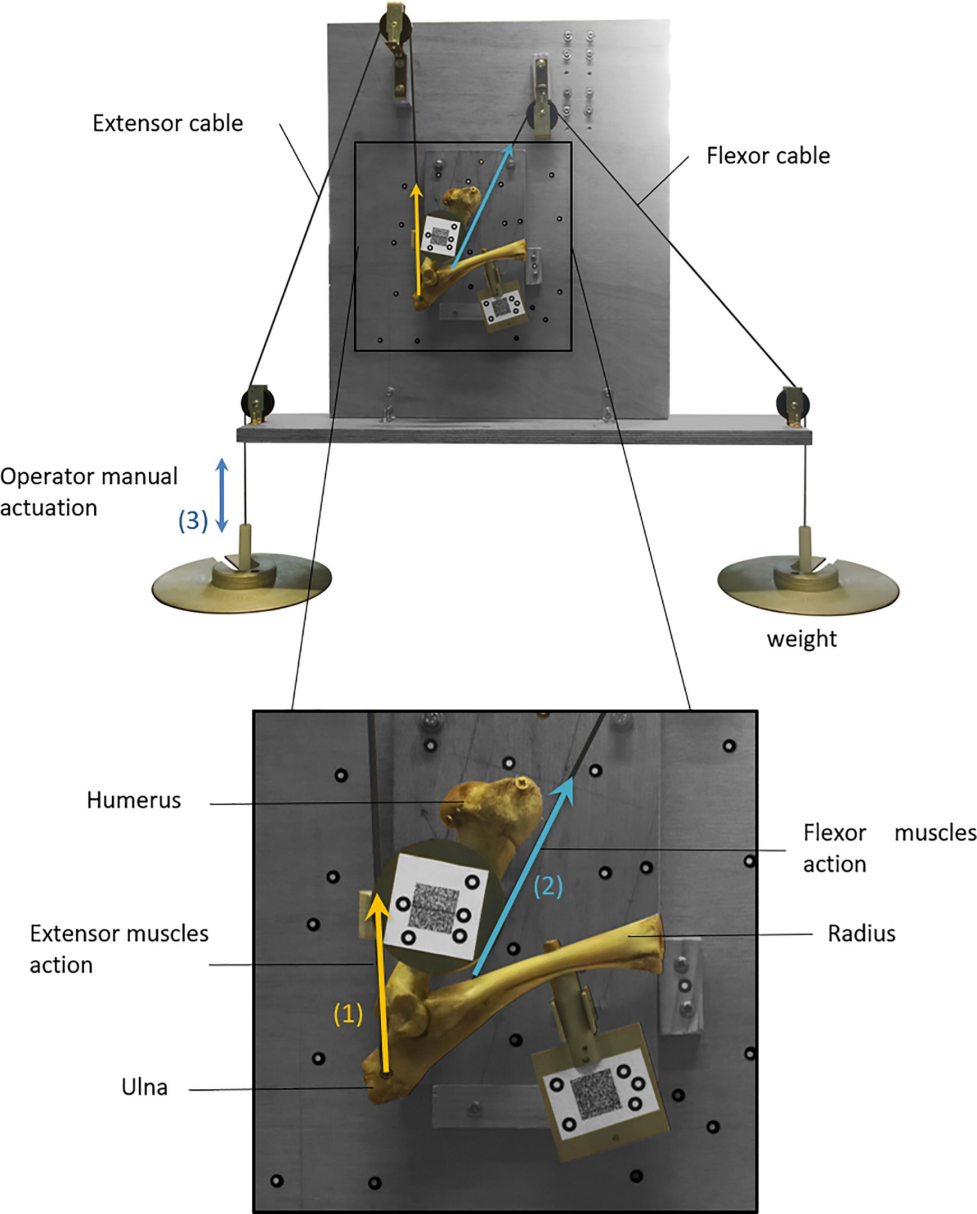
Sheep elbow test rig. (1) Extension force. (2) Flexion force. (3) Manual actuation.

This subsection describes the experimental protocol adapted in this study (*in vitro* specimen, experimental devices, experimental procedure). Five fresh cadaveric elbow specimens of healthy female Arles Merino sheep (average weight 30 kg and 1 year of age) were used for this study. No ethical approval was required because the study made use of abattoir specimens (Slaughterhouse: SA d'Exploitation de l'Abattoir Municipal de Sisteron; Location: Abattoir Municipal, 8 All des Romarins 04200 Sisteron, France). The specimens were composed of humerus, ulna and radius as well as synovial capsule and all the ligaments that ensure the cohesion of the joint. The joints were warmed to room temperature (20°C) for 30 minutes before carrying out the experiments. For initial position definition and motion capturing purposes, each specimen was equipped with two tracking clusters, which were screwed on the humerus and on the radius-ulna (see Fig 2A). The equipped elbow was set on the designed test rig (see Fig 3). The humerus was screwed on the test bench with an orientation of 15° regarding to the vertical axis (this angle is near to the humerus’ natural angle). Twenty-five measurements were performed on fresh specimens for a flexion motion from 0 to 91° (five specimens times five measurements per specimen).

To identify the equivalent insertion points of the extensor muscle group and the flexor muscle group, a dissection was done on a fresh specimen. Two cables were attached at these points (located on the radius diaphysis and the ulna olecranon) to actuate both bones. The angles of these cables were defined as the mean of the action lines of the muscles that make up the extensor and the flexor muscle group. Loads were applied at the ends of the cables holding the joint in an initial position of maximum extension (see Fig 3).

The flexion motion was generated manually performing a smooth movement for minimizing the stick-slip phenomenon and the inertial effects. Angle values were computed in terms of degrees of flexion, defined relative to a horizontal line representing zero flexion. During the motion, the trajectory of the tracking clusters attached to bones was measured using the DIC system Q400 (Dantec Dynamics, Skovlunde, Denmark, resolution of 0.002 mm). The motion was captured during 40 seconds from a measurement window of 200x200x50 mm^3^ with an acquisition frequency of 100 Hz. The measurement was repeated five times for each specimen.

In order to associate the acquired motion data with the articular geometry, bones were digitized separately (after the experiments) using a 3D optical scanner (Gom ATOS III, Braunschweig, Germany, resolution of 0.02 mm). The humerus and the radius-ulna were scanned after cutting the synovial tissue but keeping the cartilage.

To express both geometric and kinematic data in the same reference frame, the tracking clusters were provided with two different patterns: a stochastic speckle and GOM stickers (see Fig 2C). For the tracking system Q400 Dantec, two perpendicular edges of the two stochastic speckles were used to create two datums: **Ω**_**h**_ for the humerus and **Ω**_**r,i**_ for the radius-ulna. The other datum reference systems were created using three GOM stickers at distances L1 and L2 from **Ω**_**h**_ and **Ω**_**r,i**_ respectively, as shown in Fig 2C. All instantaneous positions of the reference frame **Ω**_**r,***i*_ were finally expressed in **Ω**_**h**_, defined as global frame. When expressing the acquired data in the same reference frame, it was possible to correlate the kinematic data with the geometry data of the bones.

Finally, after removing all soft tissue (including cartilage) by boiling and applying a sodium percarbonate bath, the previously described protocol was repeated for the five specimens.

### Data post-processing

**Fig 4:**
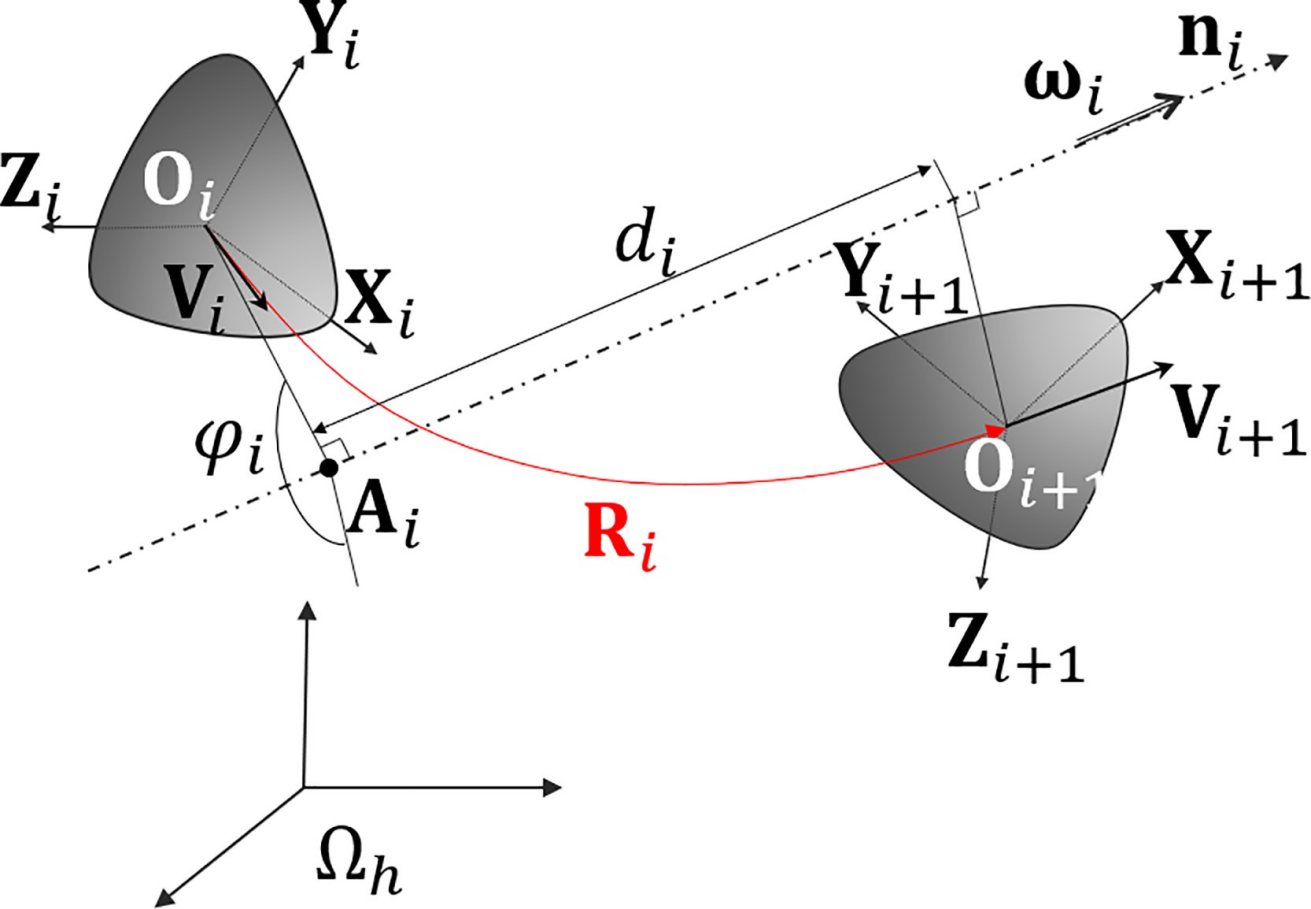
Rigid body displacement defined by the screw axis theory. **ω**_***i***_: angular velocity vector, *d*_*i*_: magnitude of the translation part of the displacement, **n**_*i*_: direction vector of the screw axis, **A**_***i***_: orthogonal projection of **O**_***i***_ on the screw axis.

This subsection presents the kinematic analysis of the sheep elbow using screw theory. This formalism allows, for all time instant *t*_*i*_, breaking down the movement between the radius-ulna and the humerus into sets of elementary displacements. These elements are: a rotation of an angle *φ*_*i*_ about a fixed axis specified by the unit vector **n**_***i***_ (n_*xi*_, n_*iy*_, n_*iz*_) and a translation of magnitude *d*_*i*_ along the same axis (see Fig 4). This was done under the hypothesis of rigid bodies.

To describe the orientation of **Ω**_**r,i**_ relative to **Ω**_**h**_, a rotation matrix **R**_***i***_ was defined between time *t*_*i*_ and *t*_*i+1*_. The Rodrigues formula (Eq 1) was used to compute the axis and the angle of the pure rotation displacement.

$$O_{i+1}=cos\varphi_{i}(O_{i})+(1-cos\varphi_{i})(O_{i}.n)n+sin\varphi_{i}(n\times O_{i})$$ (1)

The Rodrigues rotation formula (Eq 1) can also be written in matrix form (Eq 2). This allows determining *φ*_*i*_ and **n**_***i***_ directly from the matrix **R**_***i***_ after the identification of the Rodrigues parameters *S*_*i*_, *L*_*i*_, *M*_*i*_ and *N*_*i*_.

$$O_{i+1}=R_{i}O_{i}$$ (2)

where:

$$R_{i}=\left[\begin{array}{ccc} {a_{11}}_{i} & {a_{12}}_{i} & {a_{13}}_{i}\\ {a_{21}}_{i} & {a_{22}}_{i} & {a_{23}}_{i}\\ {a_{31}}_{i} & {a_{32}}_{i} & {a_{33}}_{i} \end{array}\right]=\left[\begin{array}{ccc} {S_{i}}^{2}+{L_{i}}^{2}-{M_{i}}^{2}-{N_{i}}^{2} & 2(L_{i}M_{i}-N_{i}S_{i}) & 2(L_{i}N_{i}+M_{i}S_{i})\\ 2(L_{i}M_{i}+N_{i}S_{i}) & {S_{i}}^{2}-{L_{i}}^{2}+{M_{i}}^{2}-{N_{i}}^{2} & 2(M_{i}N_{i}-L_{i}S_{i})\\ 2(L_{i}N_{i}-M_{i}S_{i}) & 2(M_{i}N_{i}+L_{i}S_{i}) & {S_{i}}^{2}-{L_{i}}^{2}-{M_{i}}^{2}+{N_{i}}^{2} \end{array}\right]$$ (3)

With:

$$S_{i}=\frac{1}{2}\sqrt{1+{a_{11}}_{i}+{a_{22}}_{i}+{a_{33}}_{i}}\;L_{i}=\frac{1}{4S_{i}}({a_{32}}_{i}-{a_{23}}_{i})$$

$$M_{i}=\frac{1}{4S_{i}}({a_{13}}_{i}-{a_{31}}_{i})\;N_{i}=\frac{1}{4S_{i}}({a_{21}}_{i}-{a_{12}}_{i})$$

Finally, we obtain:

$$\varphi_{i}=2\;arcos(S_{i})$$

$$n_{xi}=\frac{L_{i}}{sin(\frac{\varphi_{i}}{2})},\;n_{yi}=\frac{M_{i}}{sin(\frac{\varphi_{i}}{2})},\;n_{zi}=\frac{N_{i}}{sin(\frac{\varphi_{i}}{2})}$$

This formulation allows to determine the instantaneous rotation angle and the direction of the screw axis, but it does not define its location in the space. Thus, it can be done by computing the instantaneous velocity vector of a point attached to the radius-ulna bone. Using this velocity vector, the centre of instantaneous rotation can be estimated. The point **O**_*i*_ was used to compute the centre of instantaneous rotation **A**_*i*_ as shown in Eq 4.

$$O_{i}A_{i}=\frac{\omega_{i}\times V_{i}}{\omega_{i}.\omega_{i}}$$ (4)

The instantaneous velocity vector **V**_*i*_ and the angular velocity vector **ω**_*i*_ of the solid were computed by doing a four-point numerical differentiation of the displacement (Eq 5 and Eq 6).

$$V_{i}=\frac{1}{12.dt}(O_{i-2}-8.O_{i-1}+{8.O}_{i+1}-O_{i+2})$$ (5)

$$\omega_{i}=\frac{1}{12.dt}(\varphi_{i-2}-8\varphi_{i-1}+{8\varphi }_{i+1}-\varphi_{i+2})$$ (6)

The magnitude of the translation *d*_*i*_ corresponds to the projection of the vector **O**_*i*_ **O**_*i*+1_ on the instantaneous screw axis **n**_**i**_ (Eq 7).

$$d_{i}=O_{i}O_{i+1}.n_{i+1}$$ (7)

**Fig 5:**
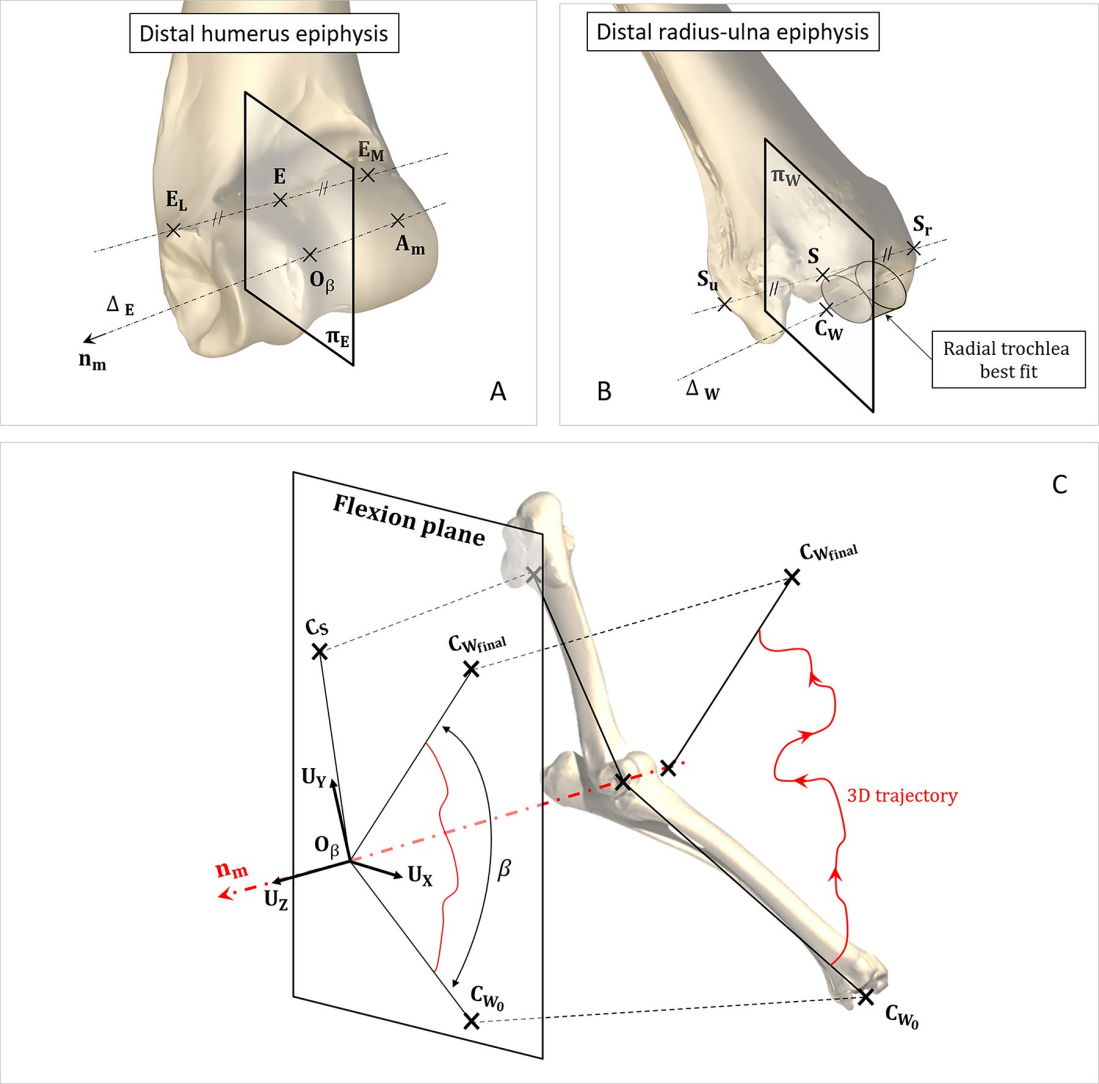
Defined anatomical reference system. (A) Geometric construction of the point **O**_**β**_, origin of the joint frame. (B) Geometric construction of the point **C**_**W**_, centre of the carpal joint. (C) Geometric construction of the elbow joint frame axis. **A**_**m**_: mean of **A**_**i**_ points, **n**_**m**_: mean flexion axis, **C**_**S**_: Centre of the a best-fitting sphere of the humeral head.

In the previous formulation, the rotation and translation displacements are expressed in a non-anatomical reference frame **Ω**_**h**_. Thus, an anatomical reference frame **Ω**_**β**_ was created following the recommendations of the ISB [34]. For the creation of this reference frame, instead of defining the flexion axis as the straight line passing through the lateral and medial epicondyles of the humerus, we used the computed flexion axis, as explained next:

- The coordinate axes of **Ω**_**β**_ were defined by means of Eq 8. This construction is depicted in Fig 5A and 5C.
  $$R_{{Ω}_{\beta }/{Ω}_{H}}=[U_{x}\;U_{Y}\;U_{Z}]$$ (8)
  $$U_{x}=\frac{A_{m}C_{S}\times n_{m}}{\left\|A_{m}C_{S}\times n_{m}\right\|}$$
  $$U_{Y}=U_{Z}\times U_{X}$$
  $$U_{Z}=n_{m}$$
  Where **C**_**S**_ is the centre of the best-fitting sphere of the humeral head, **A**_**m**_ is the mean of **A**_**i**_ points and **n**_**m**_ is the mean flexion axis.
- The origin **O**_**β**_ of **Ω**_**β**_ was defined as the intersection of the straight line Δ_E_ with the plane **π**_**E**_. The straight line Δ_E_ is defined by the vector **n**_**m**_ and the point **A**_**m**_. The plane **π**_**E**_ is defined by the vector **n**_**m**_ and the point **E**. In turn, this point is located at the middle of the line segment going through the two humeral epicondyles **E**_**M**_ and **E**_**L**_.

To define the elbow’s instantaneous flexion angle, a point **C**_**W**_ was created. **C**_**W**_ was built as the intersection of the straight line Δ_W_ with the plane **π**_**W**_ (see Fig 5B). The straight line Δ_W_ was defined as the axis of the best-fitting cylinder of the radial trochlea. The plane **π**_**W**_ was chosen normal to the line Δ_W_ and passing through point **S**. In turn, this point was located at the middle of the line segment going through the two radius and ulna styloids **S**_**r**_ and **S**_**u**_.

Once the reference frame **Ω**_**β**_ was created, the displacements measured between the bones were expressed on it. For each acquisition time, the calculated rotation was decomposed as a cascade of three simple rotations. This decomposition was done according to the Trait-Bryan sequences (pitch, roll, yaw) [35]. The obtained joint displacements, expressed in **Ω**_**β**_, were:

- Flexion movement R_Z_ in the **U**_**X**_**U**_**Y**_ plane,
- Adduction-abduction movement R_Y_ in the **U**_**Z**_**U**_**X**_ plane,
- Internal-external rotation R_X_ in the **U**_**Y**_**U**_**Z**_ plane,
- Medial-lateral translation T_Z_ normal to the **U**_**X**_**U**_**Y**_ plane,
- Proximal-distal translation T_Y_ normal to the **U**_**Z**_**U**_**X**_ plane,
- Anterior-posterior translation T_Z_ normal to the **U**_**Y**_**U**_**Z**_ plane.

RX, RY, TX, TY, TZ were expressed as function of the flexion angle as in Saul, K. et al. [36].

### Musculoskeletal inverse dynamic simulation

**Fig 6:**
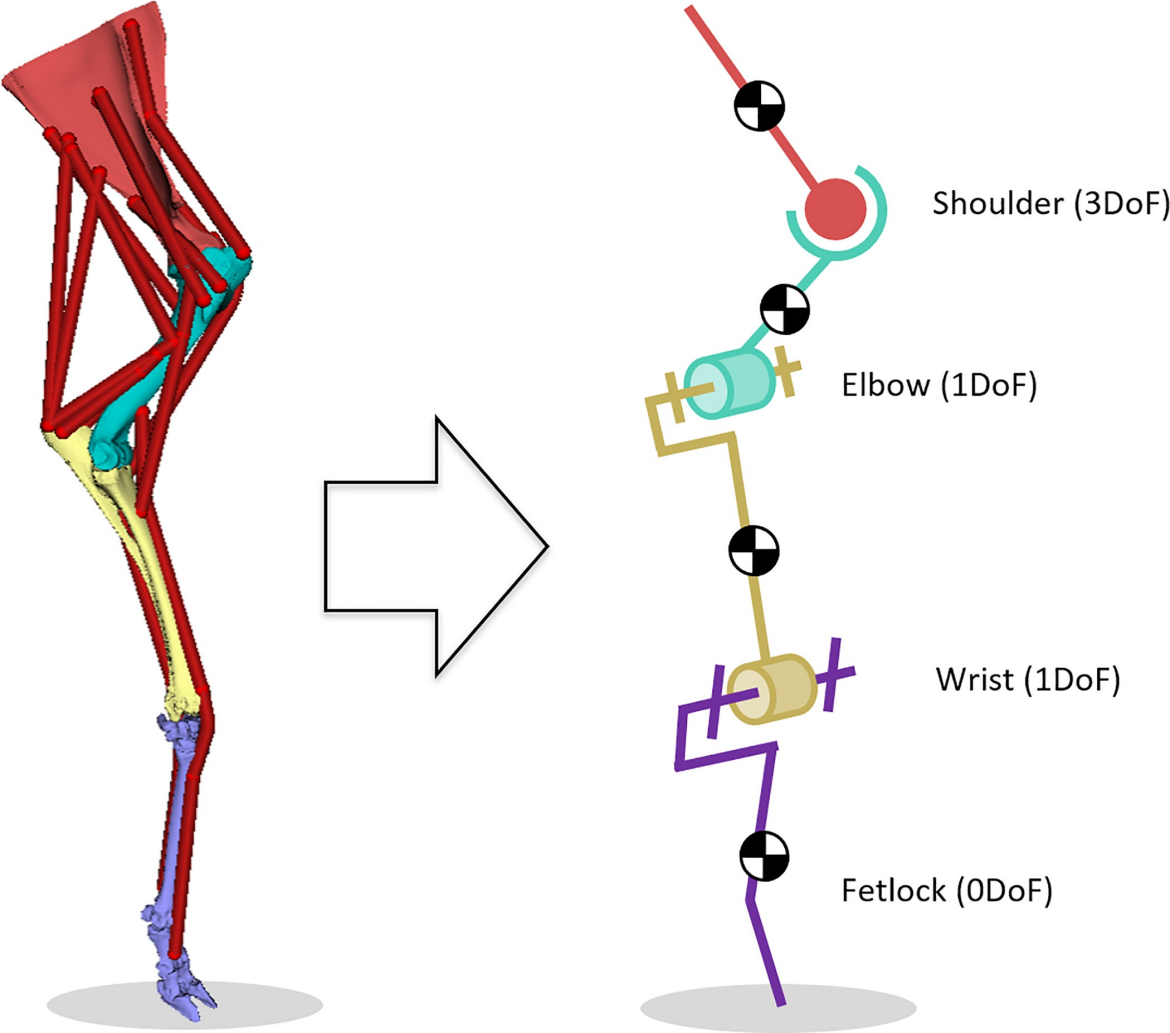
OpenSim model of the right thoracic limb of a sheep with initial elbow modelling as revolute joint.

In order to study the impact of anatomical joint simplification on the prediction of reaction forces during gait simulations, a musculoskeletal model of the right thoracic limb of a sheep was used (Fig 6). The model was composed by 4 segments and actuated by 16 Hill-type muscle-tendon units. Each extensor and flexor group was treated as a whole unit as no DoFs at the digital joints were considered. Sheep gait data was adopted from [37]. For the adopted gait trail, muscle forces were calculated using the static optimization algorithm implemented in OpenSim [38]. This algorithm resolves the net joint moments into individual muscle forces while minimizing squared muscle activations as objective function [39]. With the same software tool, joint reaction analyses were performed to estimate the loads acting at the elbow joint. We compared the simulation results of the three models mentioned below. For each model, a new muscle solution was generated and a new joint reaction analysis was performed:

1. In the first model, the shoulder was represented by a ball-and-socket joint, while the elbow and carpal joints as revolute joints. The geometric parameters of these joints were determined by fitting analytical surfaces (sphere and cylinders, respectively) to the articular surfaces.
2. In the second model, the rotation axis of the revolute joint representing the elbow was redefined according to the one obtained from the motion analysis **Ω**_**β**_.
3. In the last model, the elbow was modelled as a complex joint including the measured displacements R_X_, R_Y_, T_X_, T_Y_, T_Z_ as function of the flexion angle.

## Results

### Joint kinematics

**Fig 7:**
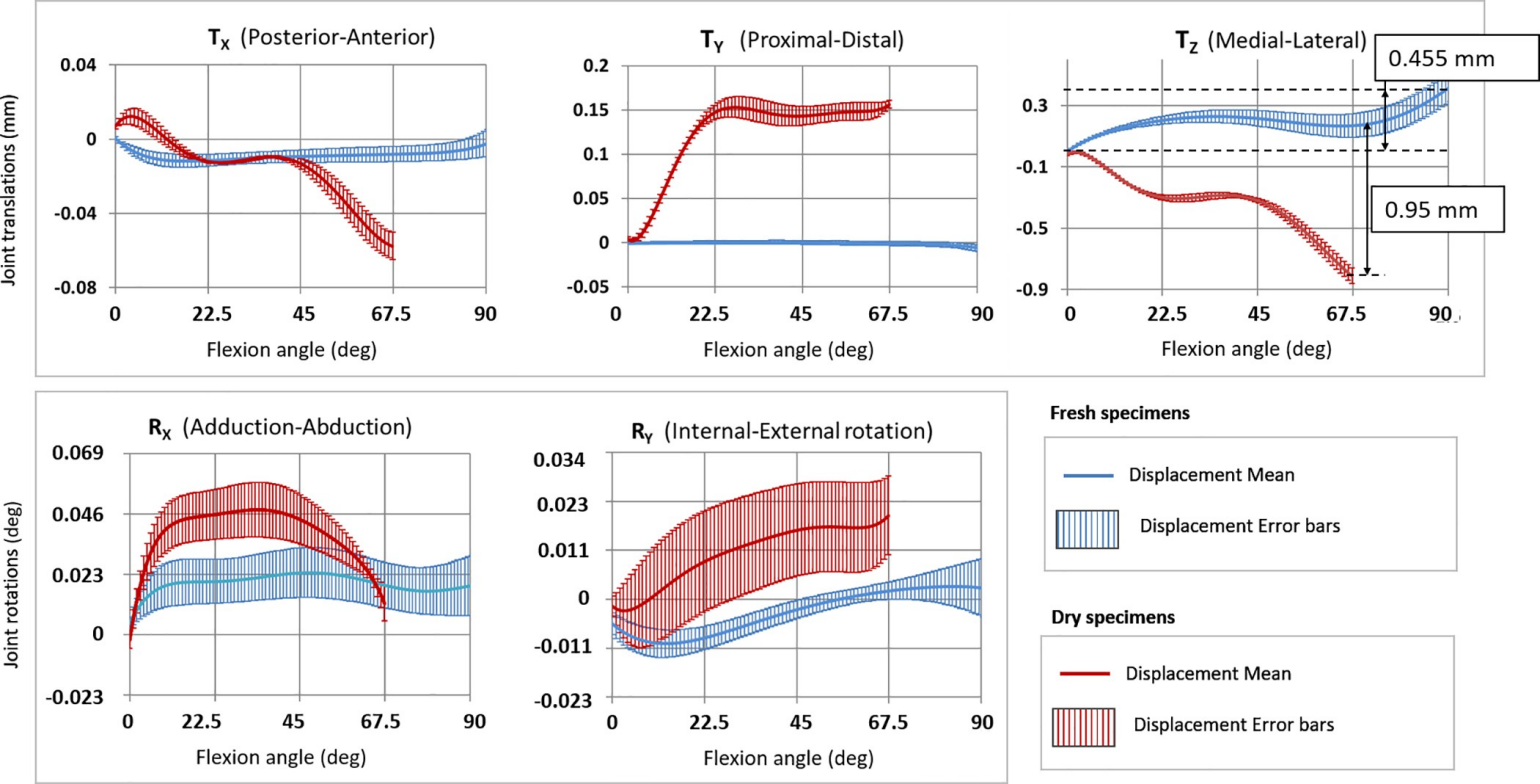
Decomposed elbow displacements. Mean and error bar of the 5 displacements as function of the flexion angle for fresh specimens with ligaments and dry bone specimens.

The maximum measurement uncertainty obtained during the experiment was 0.012 mm for the DIC system and 0.02 mm for the 3D scanner. For each acquisition time, the mean values of the calculated displacements (R_X_, R_Y_, T_X_, T_Y_, T_Z_) are shown in Fig 7 with their error bars. These error bars were computed with a critical value t = 2.064 considering a Student's t distribution with 24 degrees of freedom (number of measurements less one) for a two-tailed level of significance of 5%.

The displacement characterization curves emerging from the set of fresh specimens are shown in Fig 7 (blue colour). The obtained medial-lateral translation (Tz) ranged from 0.002 (± 0.025) to 0.457 mm (± 0.119). Posterior-anterior (Tx) and proximal-distal (Ty) translations ranged from -0.012 (± 0.026) to 0.001 mm (± 0.025) and from -0.008 mm (± 0.026) to 0 mm (± 0.022), respectively. Adduction-abduction (Rx) and internal-external (Ry) rotations ranged from 4.41e-3° to 23.5e-3° and from -10.3e-3° to 3.04e-3°.

The displacements of the dry bone specimens are plotted in red colour in Fig 7. In these experiments, the absence of the synovial capsule and ligaments limited the range of the flexion motion. The obtained medial-lateral translations (Tz) ranged from -0.81 (± 0.029) to -0.017 mm (± 0.2). Posterior-anterior (Tx) and proximal-distal (Ty) translations ranged from -0.057 mm (± 0.032) to 0.012 (± 0.044) and from 0.004 mm (± 0.036) to 0.153 mm (± 0.047), respectively. Adduction-abduction (Rx) and internal-external (Ry) rotations ranged from 0 to 47.50e-3° and from -2.75e-3° to 19.6e-3°.

When comparing the results from fresh and dry specimens, we observed significant differences for all displacements (see Fig 7). The maximum difference between fresh and dry specimens was noticed in the medial-lateral translations (Tz) when the flexion angle was 67.5°. Regarding the uncertainty of the measurement process, only this displacement was significant.

### Flexion axis location

**Fig 8:**
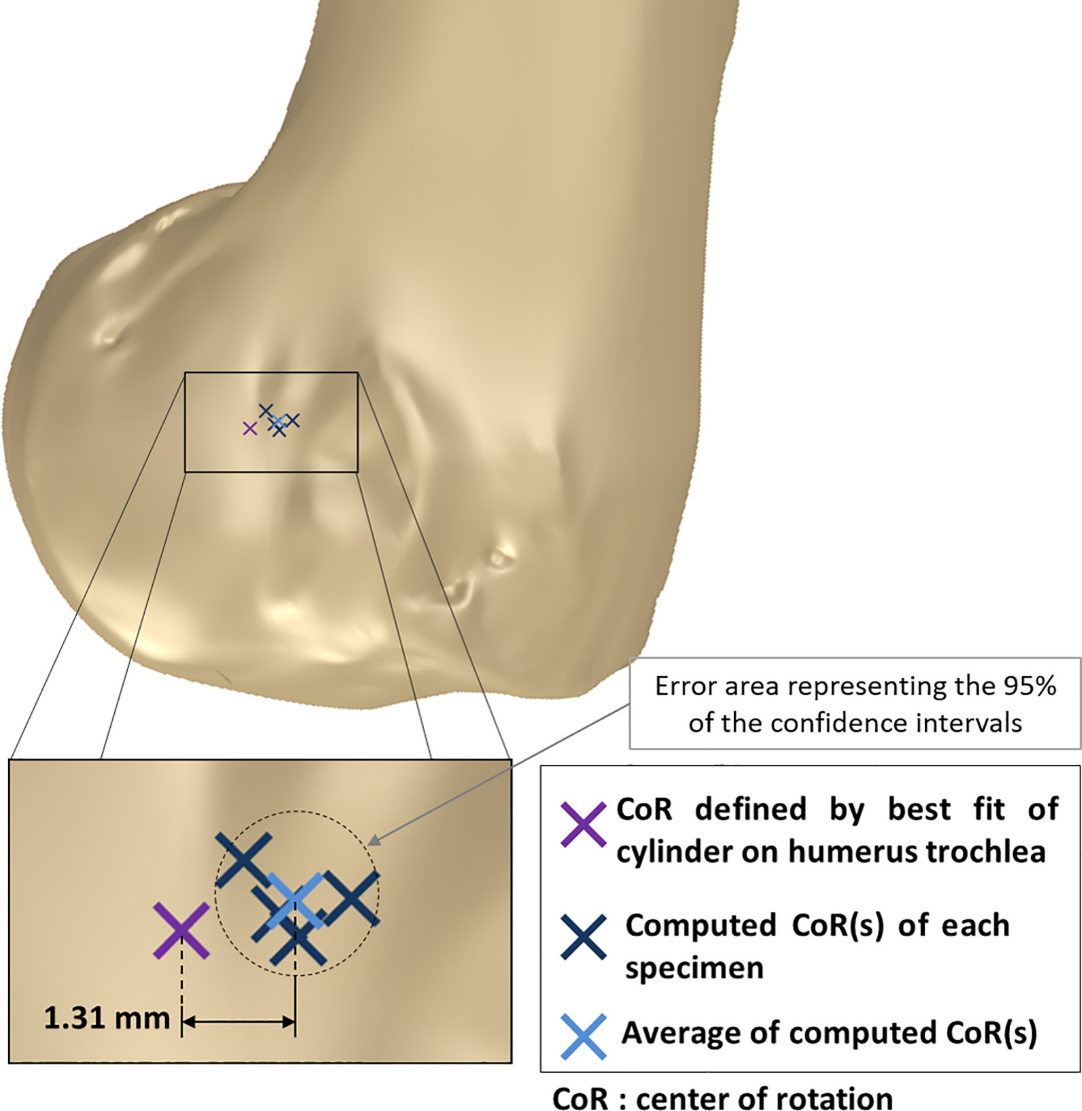
Mean rotation axis location on distal humerus epiphysis sagittal view.

We compared the location of the mean axis of rotation obtained from the previous kinematic analysis with the rotation axis defined by the best-fitting cylinder of the humeral trochlea (method used by [7,9]). These results are shown in Fig 8.

We found a mean variation of the axis location of 1.31 mm (±0.412). This analysis was done in the **U**_**X**_**U**_**Y**_ plane considering the intersection point of the best-fitting cylinder axis with this reference plane and the point **O**_**𝛃**_. The angular variation between these axes is of 5.11° (±2.25).

### Joint reaction forces

**Fig 9:**
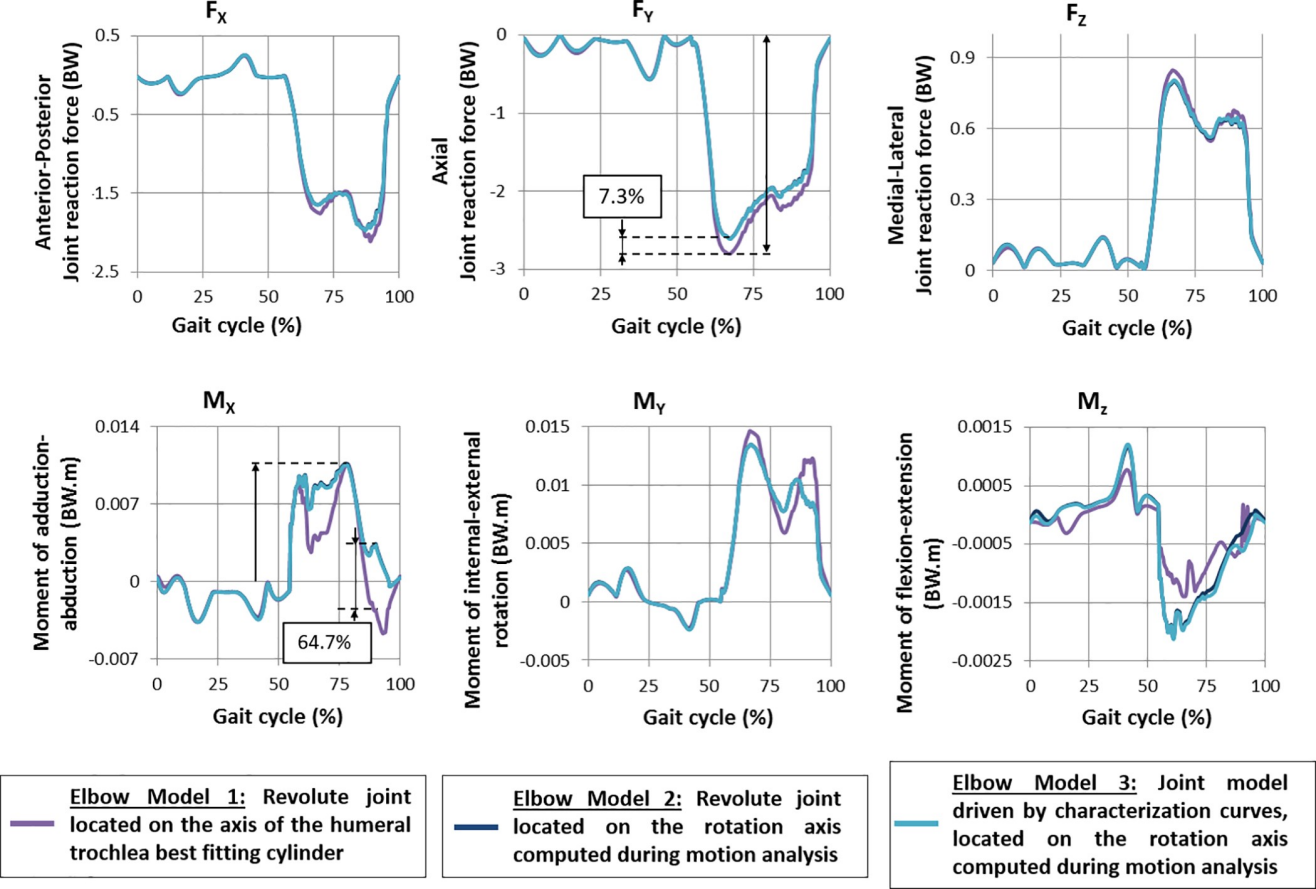
Simulated reaction forces at the elbow.

Joint reaction forces at the elbow joint were computed from the three musculoskeletal models (Fig 9). Forces (Fx, Fy, Fz) and moments (Mx, My, Mz) are plotted as function of the percentage of the gait cycle. These values were normalized against body weight (BW). The results of model 1 (revolute joint located on the axis of the humeral trochlea best fitting cylinder) is plotted in purple. In dark blue, the results of the model 2 (revolute joint located on the mean flexion axis) are represented. The results coming from model 3 (revolute joint combined with a medial-lateral translation) are plotted in blue sky.

Regarding joint forces, a maximum difference between models 1 and 2 of 7.3% of the peak force in Fy can be noticed. In joint moments, a difference of 64.7% of peak moment was obtained in Mx. Between the results of models 2 and 3, the observed differences are equal to 0.7% of peak joint forces (Fy) and 9.8% of peak joint moment (Mx) for the maximum values.

## Discussion

To the best of our knowledge, this article proposes the first 6 DoF kinematics analysis of the elbow of a quadruped animal. For this analysis, we conducted *in vitro* experiments over sheep elbow specimens performing extension–flexion motion. Looking for a high measuring precision, we used a DIC system for capturing bones motion. The fact of measuring directly over the bones avoided issues induced by soft tissue artifacts. This measured kinematics was implemented in a musculoskeletal model of a sheep forelimb to evaluate its impact in the prediction of joint reaction forces.

The motion analysis showed that only the medial-lateral translation is significant to the measuring uncertainty of the experiment. This translation assimilates the sheep elbow to a screw joint instead of a revolute joint. These results are in contrast with the findings of [40], in which no significant translations between the humerus and the radio-ulna were observed in dogs. This can be explained by the fact that the measurement uncertainty of their motion capture system was too high regarding the order of magnitude of these displacements. This medial-lateral translation was previously reported in human elbows [41]; however, the kinematic behaviour of human and sheep elbow is scarcely comparable because of their anatomical and functional differences (lifestyle, angles of motion, pronation-supination).

From the results of our inverse dynamic simulation, we could observe that the inclusion of the medial-lateral translation to the model made up with the mean flexion axis does not affect the prediction of joint reaction forces. Therefore, from a dynamic point of view, the sheep elbow can be assimilated to a revolute joint. These results agree with the conclusions of Martelli S. [42]. Nevertheless, in the case of small-scale analysis (i.e. contact analysis), the complete model including the axial translation should be considered [10]. Ongoing work is focusing on understanding how the observed kinematics influences the contact mechanics of the joint.

We found a geometrical difference between the axis of the best fitting cylinder and the mean flexion axis (derived from the motion analysis) of fresh specimens. This can be explained by the fact that the best fitting cylinder was calculated from the digitized geometry of the humerus when it was not subjected to loads. In contrast, during the conducted experiments, the cartilage of the humerus was certainly deformed due to the load, which can partially explain this difference. This geometrical difference finally impacts the prediction of joint reactions. These differences could be due by the change of lever arm due to the different location of the flexion axis. The results obtained from our inverse dynamic simulations are in the same order of magnitude as those reported by others [1,3,4]. These studies were also performed in sheep models, although they analysed the dynamic behaviour of the hind limb.

In comparison with fresh specimens, the experiments with dry bones provided different kinematic behaviour. This can certainly be explained by the absence of cartilage and ligaments, which induces a large gap in the joint and affects the continuity of the contact between the articular surfaces. The lack of ligaments affects the joint’s stability as highlighted in [43,44] and allows larger displacements of the bones. Thus, under the conditions of the conducted experiments, it is not possible to conclude if joint kinematics of dry bone specimens could be representative of those of fresh specimens. Therefore, further work is needed on the design of a new experimental protocol in which a continuous contact between the articular surfaces of the dry bone specimens be guaranteed.

This study has several limitations. First, the experimental protocol was conducted *in vitro*. It implies a non-natural joint actuation, which could potentially impact the joint kinematic and dynamic behaviour. Furthermore, the absence of muscles on the specimen itself can affect the joint kinematics due to reduction of muscle passive holding. Second, the inter-specimen comparison and the associated reference frame matching inevitably leads to errors. Finally, for the inverse dynamic simulation, several considerations were made: bones were considered as rigid bodies, segments' anthropometric properties were considered not time-dependent, biological joints were modelled as frictionless joints and ground reaction forces were applied on the distal phalanx. Additionally, the same data set of ground reaction forces was used for all musculoskeletal simulations. Work is ongoing on developing an automated test rig for an interspecies comparison of the kinematic behaviour of the elbow.

In conclusion, this study showed that the sheep elbow joint has a more complex kinematics than a revolute joint. Besides, the mean flexion axis computed during experiments and the one derived from articular surfaces best fit do not have the same location. However, from a dynamic point view, the sheep elbow could be considered as a revolute joint.
